# Midwives' Role in Providing Nutrition Advice during Pregnancy: Meeting the Challenges? A Qualitative Study

**DOI:** 10.1155/2017/7698510

**Published:** 2017-07-02

**Authors:** Jamila Arrish, Heather Yeatman, Moira Williamson

**Affiliations:** ^1^School of Health and Society, Faculty of Social Sciences, University of Wollongong, Northfields Avenue, Wollongong, NSW 2522, Australia; ^2^School of Nursing, Faculty of Science, Medicine & Health, University of Wollongong, Northfields Avenue, Wollongong, NSW 2522, Australia; ^3^School of Nursing and Midwifery, Higher Education Division, Central Queensland University, 90 Goodchap Street, Noosaville, QLD 4566, Australia

## Abstract

This study explored the Australian midwives' role in the provision of nutrition advice. Little is known about their perceptions of this role, the influence of the model of care, and the barriers and facilitators that may influence them providing quality nutrition advice to pregnant women. Semistructured telephone interviews were undertaken with a subsample (*n* = 16) of the members of the Australian College of Midwives who participated in an online survey about midwives' nutrition knowledge, attitudes, and their confidence in providing nutrition advice during pregnancy. Thematic descriptive analysis was used to analyse the data. Midwives believed they have a vital role in providing nutrition advice to pregnant women in the context of health promotion. However, this was not reflected in the advice many of them provided, which in many accounts was passive and medically directed. The extent and efficacy of their role appear to be challenged by many structural barriers. Midwives suggested facilitators that may assist in overcoming these challenges. Midwives need assistance, support, and guidance to provide holistic nutrition advice that assists women to achieve healthy pregnancies. A collaborative approach between midwifery bodies, nutrition and education experts, and maternity care services may provide an effective way forward.

## 1. Introduction

The short and long term impacts of maternal diet on the health of the mother and the foetus are widely documented [1, 2]. Pregnant women's dietary behaviour is influenced by interpersonal, institutional, and community factors [3]. Nutrition knowledge is one of those numerous factors that may affect women's diet [3] and pregnant women are perceived to be more receptive of nutrition information during pregnancy [4]. Nutrition education during pregnancy has been linked to positive maternal and infant outcomes [5], especially among overweight and obese women [6]. In spite of these positive links, the provision of nutrition advice by antenatal care providers is not common practice [7]. In the literature, this lack of engagement with women in discussing their diet has been attributed to health care providers being challenged by factors such as limited time, resources, and education [8].

Midwives are health care professionals perceived to be in a unique position to provide nutrition advice to pregnant women due to their usual contact with the women via antenatal appointments. Moreover, health promotion and education are considered among the most important activities that midwives perform with pregnant women as advocates for health and wellbeing rather than managers of diseases [9]. However, studies in the United Kingdom (UK) [10] and Sweden [11] reported that midwives struggle to provide dietary advice, especially in the context of health promotion and on challenging issues such as obesity, despite acknowledging it as part of their role [10].

A recent Australian quantitative study reported that the majority of the midwives believed that their role in providing nutrition advice is significant and the majority reported providing nutrition advice to pregnant women. However, some midwives provided written comments specifying barriers such as lack of time, resources, and the model of care currently utilised as affecting their provision of such advice [12].

This study aimed to gain further understanding of midwives' perceptions of their role, particularly the effect of the model of care on the way they provide nutrition advice, the barriers that hinder their role, and the facilitators that may help them to provide better nutrition advice to pregnant women. These insights will contribute to pregnant women receiving quality nutrition advice and support to help them make informed decisions about their diets.

## 2. Methods

### 2.1. Design

A qualitative descriptive approach was undertaken to gain an in-depth understanding of midwives' perception of the importance of nutrition, how they learnt about nutrition, and how they educate women. The approach is useful when the aim of the research is to describe participants' experiences and perceptions regarding a particular topic, as is the aim of this research [13]. In the process of analysing the data and presenting them, there is rich and straightforward description of the participants' experiences or related events [13]. The analysis in this approach allows for reasonable interpretation but the researcher stays close to the data and participants' own language [13]. Semistructured interviews were employed. The interviews were flexible in style and structured around an interview guide and also included other probing questions that may arise during the interview. The questions were open-ended in nature, allowing respondents to give their answers in their own words and to express their ideas and opinions. The purpose of a semistructured interview is to generate rich and candid data that lead to a deeper understanding of an issue [14].

### 2.2. Sampling and Participants

The participants were a convenience subsample of the members of the Australian College of Midwives (ACM) who participated in an online survey about midwives' nutrition knowledge, attitudes, and their confidence in providing nutrition advice during pregnancy in 2012 [12].

### 2.3. Recruitment

In the participant information page of the midwives' online survey, an introduction about follow-up interviews was provided and a note was also included at the end of the survey. Midwives were directed to a separate page to indicate their consent to be contacted for the follow-up interview and to provide their contact details (i.e., names and email addresses). The intention was to gain the views of midwives across different ages and experiences. Therefore, the participants were asked to indicate their age category (i.e., younger than 35 or older than 35) and include their years of experience. The invitations for the interviews (including the participation information sheet and the consent form) were formally sent in October 2012 to the midwives who had indicated their consent to being contacted for the follow-up. The participant information sheet explained the aim of the study, its significance, what the participants will do, the approximate time that will be taken for the interviews, and the participants' right to refuse to participate or withdraw from the study with their data at any time without any effect on their relationship with the University of Wollongong or their place of employment. The participants were offered the opportunity to review their transcripts if they wanted. How the data will be disseminated was also explained. The participants were also given the details of the ethics committee of the university who they could approach with any concerns. They were given the opportunity to ask any questions at any stage. Reminders to nonrespondents were sent in November 2012 and January 2013. Participants who returned their signed consent forms were sent a form to indicate their preferred option for the interview (Skype or telephone), provide their phone if they chose the telephone or their Skype account's details if they chose Skype, and provide their preferred dates for the interview.

### 2.4. Data Collection

The primary researcher (JA) conducted the semistructured telephone interviews between October 2012 and March 2013. The interviews were audio-recorded. The length of the interviews averaged 21 minutes. The main questions that were used as a guide during the interviews were as follows:

1. Can you please describe the model of care you follow/practise as a midwife?
2. I would be interested to hear your views of how food selection and nutrition during pregnancy could influence pregnancy outcomes.
3. What are the most important food issues that you usually discuss with pregnant women during their antenatal visits? What are the issues that should be discussed from your point of view?
4. In what ways do you think your model of care affects the way you provide nutrition advice?
5. Please describe for me the role you think midwives should have in regard to providing food-related or nutrition advice to women during pregnancy?
  - What preparation do you think midwives receive to provide such advice?
  - What are the barriers that midwives encounter in relation to providing such advice?
  - What guidelines or sources of information are available to midwives relating to providing nutrition information to pregnant women?
6. What might assist midwives to provide better services for pregnant women in regard to food-related or nutrition advice?
7. Would you like to add anything else?

Further questions or prompts derived from the interviews were used as relevant. The interview guide was developed by JA. The development of the guide was informed by the literature and the aim to deepen the understanding of the findings of the survey. The coresearchers, two experts (midwifery and public health), and a dietitian reviewed the interview guide to determine its relevance to achieve the aims of the study.

### 2.5. Data Analysis

All interviews were transcribed verbatim by a professional transcriber. The interviews were then checked for accuracy by the primary researcher (JA). The Software QSR International's NVivo11 was used to manage the data. Thematic descriptive analysis was used [15]. The transcripts were read and reread by JA to ensure familiarity with the data and the audio recordings were referred to frequently to interpret the answers in their actual context. Open coding of the transcripts was undertaken inductively. Two researchers (JA and MW) undertook independent coding to ensure agreement of coding and establish validity and rigor. When disagreement existed, the coding was discussed between the researchers until consensus was reached. JA completed the analysis, organised the descriptive themes, and verified them with all coresearchers.

### 2.6. Ethical Considerations

The University of Wollongong Health and Medical Human Research Ethics Committee approved the study (HE12/009). In the participation information page of the online survey, the interested midwives were assured that their personal details would be secure if they decided to participate and their anonymity would be reserved. All participating midwives sent their signed consent forms either by email or through the post via the means of prepaid envelopes. A verbal confirmation was also indicated at the beginning of the interviews.

## 3. Findings 

### 3.1. Participants

Fifty-two midwives consented to be contacted for the interviews and provided their contact details. Nineteen midwives signed their consent forms and sent them back but three midwives did not send the forms of dates and other details despite multiple contacts. Hence 16 midwives (all of which were female) participated in the study and no selection criteria were applied. All, but one, midwives were older than 35 years of age. The participants' years of experience ranged from 2 years to 37 years.  Table 1 shows the models of care that the midwives specified they were employed in. The midwives worked in a variety of settings including public hospitals, private hospitals, private practice, hospital and community antenatal clinics, and birth centres. Some midwives had one role, while others had more than one role. For some midwives, antenatal care was their main area of work and expertise while others rotated through different areas (i.e., birthing suite and postnatal care). Some midwives worked individually while others worked in teams. Seven of the midwives specified that their models provided continuity of care, while eight midwives did not work in continuity of care models but rather provided fragmented care. One midwife had two roles providing both continuity of care and fragmented care.

### 3.2. Perceptions of Midwives' Role in Providing Nutrition Advice

The majority of the participants perceived the provision of nutrition advice to pregnant women as a vital part of the midwives' role. They considered that midwives have a unique opportunity to do so as they are the health professionals who have the most contact with pregnant women. Some midwives indicated that this opportunity would be greater in a midwifery-led model of care.

I think midwives are health promoters…. Health educators…. I think midwives have a unique opportunity to engage with women at that level and if you're providing, if you're a continuity of carer, so that means that a woman's having care from a known midwife then basically throughout the pregnancy you can establish a relationship of trust and respect, and then you can work with the women more closely with regards to their specific lives.... support her to provide good nutrition for her family. (Midwife 1)

There was a general consensus that midwives are the “first port of call” and not the experts, so they provide general or basic nutrition advice within the context of health promotion and primary health care. Providing nutrition advice for specific or complex issues is the role of the experts, “dietitians.”

I think midwives are the first port of call…. I think the midwives can provide the basic information and if there is a particular problem or if there's a lady [who] has got a particular dietary problem that's when you would refer to a specialist, a dietitian. (Midwife 2)

Despite believing in their role in providing nutrition advice, some midwives pointed out that the extent of this role either depends on the model of care or practice setting or is largely restricted by many barriers such as time and model of care.

Well, in an ideal world pregnant women in Australia would have midwifery-led care… from conception right through pregnancy til 6 weeks afterwards…. So it'd just be a gradual osmosis of information throughout the pregnancy on diet and, you know, alcohol and nicotine and it'd just be gradual. (Midwife 14)

### 3.3. Effect of Model of Care on the Provision of Nutrition Advice

#### 3.3.1. Continuity of Care versus Fragmented Care

Midwives in midwifery-led continuity of care models believed that the best advantage of their model of care was the ability to build a relationship with the women based on trust, respect, and confidence. In this context, the woman would feel comfortable talking about her dietary behaviour with the midwife and the midwife would feel comfortable approaching the woman to provide woman-centred advice. They also thought that continuity of care models allowed more time for the provision of verbal, gradual, and deeper nutrition information that could be absorbed by the woman.

…I've got a lot of time to spend with them…. I get the feeling that they trust me and that they would trust my advice…. Because I see them regularly, I can give them just bite size amounts of information which can be absorbed quite well rather than great lumps of stuff. Also most of it's a verbal education therefore there's not all the written stuff so I'm not relying on their literacy skills. (Midwife 12, community-based/continuity of care model)

One midwife thought of nutrition as a means to help improve birth outcomes for her home birth women.

Well, it's very important to me as a practitioner… that I've done everything that I can antenatally to get the best outcome at the birth because most of my clients are home birthers…. So I want them to be really healthy, really well nourished, really strong, prepared for labour, so everything goes as smoothly as possible. (Midwife 7, midwifery-led care/continuity of care)

Some midwives discussed customising their advice and messages according to their perceptions or assumptions of the women they took care of.

I think in that context of the birth centre generally women are well educated. It's really just reinforcing what a lot of women know…. (Midwife 13, group practice/birth centre/continuity of care)

Midwives employed in public hospitals or the private sector in traditional fragmented models of care and not continuity of care midwifery models, on the other hand, felt affected by many factors in the way they provided nutrition advice. Most signalled that their model of care did not allow enough time, opportunities, or early involvement to build a relationship with the women and provide effective education.

…if you know that you're going to see them a few more times, then you can actually build a relationship and then start to build up your rapport and their trust in you to the point where they're going to be willing to accept what you have to tell them. (Midwife 5, hospital/rotating through all areas and does antenatal clinic/fragmented care)

One midwife working with an obstetrician in a private hospital mentioned being restricted by his practice and beliefs in the way she advised women about nutrition. She also thought that she would be more forthcoming in discussing nutrition if she was working in the public sector.

…[the obstetrician] was popular…I had to be mindful of what he felt and the way he worked and therefore I didn't want to upset anybody commenting on their weight or giving them advice if it was unasked for…. So if somebody asked me or made a comment about being overweight, what can I do about it, then I would offer it, but I didn't volunteer it. (Midwife 9, private practice for an obstetrician/fragmented care)

A few participants presented their model of care as a holistic model looking at the women “as a whole.” They expressed the view that the health of the women is affected by many factors, and nutrition can affect the woman's health and that of her baby and family in many ways during and beyond pregnancy. In comparison to other health professionals such as doctors and dietitians, one midwife thought that midwives generally focused more on nutrition education compared to doctors, while another acknowledged that her technical knowledge is limited compared to a dietitian but this suited the low level of literacy of the women she worked with.

…I think, in some ways, the fact that I don't have a lot of the really technical scientific knowledge around nutrition maybe doesn't actually matter that much in the sense that for many of my clients their own…literacy skills can be quite limited and their language skills can be quite limited. So actually keeping things really, really succinct and simple is quite important in providing meaningful education…(Midwife 4, community-based/continuity of care model)

All midwives agreed on the importance of nutrition during pregnancy and were generally aware that it has impacts on the health of the mother and the baby. However, not all midwives could name specific pregnancy outcomes affected by nutrition behaviour and food selection of pregnant women.

Some midwives viewed nutrition as the “first medicine,” so they tended to favour food over supplements when discussing nutrition issues with pregnant women, especially as it was believed that women were commonly more receptive of nutrition messages while pregnant.

So in terms of just women in pregnancy I think our team's attitude was that nutrition should be the first medicine…. So then if you're looking at say a woman who has low-ish iron stores at the beginning of pregnancy then we're looking at foods to increase her iron source…we're also looking at micronutrients in food as opposed to going to the chemist and buying a tablet. (Midwife 1)

Poor nutrition during nutrition was believed to be linked to outcomes such as preterm birth, large babies, postpartum haemorrhage, overweight, obesity, neural tube defects, anaemia, gestational diabetes, limited choice of model of care, miscarriage or stillbirth due to listeria, and for the baby to be prone to chronic diseases later in life. Good nutrition on the other hand was linked to better health for the mother and the baby, managing pregnancy outcomes, and preventing complications such as gestational diabetes.

Nutrition was not routinely discussed, especially in a comprehensive manner. All midwives discussed nutrition issues on some level; however, on closer examination many midwives used generalised or passive approaches, where they considered the provision of written information sufficient, especially in terms of healthy eating advice for women they perceived or assumed to be healthy women.

So in terms of information about nutrition on their booking, we would give women information about nutrition on booking, a written pamphlet. It was generated from Better Health; Victoria has a Better Health channel which gave a good overview. (Midwife 1)

There was more focus on certain aspects of nutrition during pregnancy, especially those related to biomedical knowledge and blood tests when suspecting a deficiency or managing an issue. This approach was in contrast to midwives' perception of their role in providing nutrition advice as health promoters and followers of a holistic approach.

…I suppose the main one was in the birth centre context that we tend to have more discussion over is anaemia. So the influence is the woman's iron is staying down low, of course you've got the potential for either being affected if, you know, they have a normal blood loss or the risk of you know, postpartum haemorrhage, recovery, long term breastfeeding, you know, so we talk in those contexts if we find that someone's iron is down low…The other dietary aspects, so within the anaemia realm you know, you're looking at women who maybe are vegetarian so making sure women have a very well rounded base with a vegetarian and supplements, so all of those things. (Midwife 13)

Some midwives had an active and individualised approach to discussing nutrition with the women, where they provided verbal advice and did not merely depend on written information. These midwives reported they discussed the woman's diet and provided advice accordingly.

While some midwives focused on certain nutrition aspects relevant to the women they provided care for (e.g., alcohol), others reported they abstained from discussing some aspects of nutrition (e.g., listeria and folic acid, or reducing consumption of caffeinated drinks and junk food). This was based on their assumptions of women's prior knowledge of the topic. Midwives made assumptions about a woman's status and attitudes (i.e., perceiving the woman to be motivated, well, or educated). Also, as a result of late professional encounters with the women, midwives reported assuming they were already informed (either the woman had sought information themselves or had received information earlier from other health professionals).

In terms of weight issues, not all midwives mentioned discussing weight with the women, and some among those who reported doing so did not address it in-depth or directly. Among the reasons mentioned was avoidance of causing worry to the women and trying to minimise the guilt around weight gain.

…I guess I try and minimise the guilt factor in how much weight they put on because there's such a wide range and as long as they're healthy. (Midwife 2)

Some midwives indicated that if they had more time, knowledge, and resources, they would like to focus more on promoting healthy eating in general and specific aspects related to pregnancy (e.g., obesity and diet for women from different cultural backgrounds). They indicated they believed that providing adequate support to women to improve their health was important as pregnancy is a great time to do so.

### 3.4. Preparation to Provide Nutrition Advice

Half of the midwives believed that the coverage of nutrition during pregnancy within their midwifery education was nonexistent or limited. If they recalled having received such pregnancy nutrition education, they remembered it as being mainly focused on basics and lacking variation. Some of those midwives indicated that they gained most of their knowledge through work experience and communicating with other health professionals such as dietitians, a process which was perceived to be more difficult than being taught in a formal manner.

Some midwives reported receiving nutrition education during their initial education, either in nursing or midwifery education. However, nutrition education was perceived as like any other area where learning is life-long and it needed continuing education.

…I mean I've been a midwife for a long time and I also did general nursing first. So…in my general nursing there was fairly good,…education on nutrition but it's a long time ago. And then it's really just what I've read…and I think I've got knowledge gaps as well, so…I think all midwives should have ongoing education in nutrition because,…things change and we learn new information and that's not necessarily,…spread to the people working in the work place. (Midwife 10)

Most of the midwives in this study were of mature age and some mentioned being trained through hospitals. Many acknowledged that they were not aware of the nutrition education provided in Australian midwifery courses offered at universities but hoped that there is inclusion of current nutrition-related issues and specific issues such as food intolerances and special diets. However, one midwife who was trained through hospitals had also done a Bachelor of Midwifery in the university system. She undertook a designated unit on nutrition but reported it had focused more on breastfeeding than on maternal nutrition.

There was a general view that Australian midwives do not receive adequate formal nutrition continuing education or support while practising, perceiving this as a reflection of nutrition being a neglected area. Midwives especially remarked that there was a lack of continuing education activities or opportunities for education about nutrition during pregnancy in the broad sense. Nutrition issues were reported to be threaded into other issues rather than presented separately at conferences or during in-service education. The focus of the limited education that is available was perceived to be in relation to particular groups or issues (such as obesity), reflecting issues with a high public focus.

…in recent years, with the obesity issue it's come back into the focus a whole lot more now. I mean there was a big focus a few years ago with listeria…there was a big push on…providing a whole list of foods to be avoided and then that sort of…went off the radar a bit and then it's come back in again…it's very limited, extremely limited. (Midwife 8)

Midwives considered self-directed learning (SDL) an essential part of their job and it was their responsibility to update their nutrition knowledge and practice. However, it was commented upon that lack of mandatory education might have led to more reliance on self-directed learning. This was problematic as it was believed that midwives would need to be highly motivated, interested, or working mostly in antenatal care to seek continuing education in nutrition.

…as a midwife in Australia, I don't know that a lot of time is given to nutrition in pregnancy… it's behoven on the midwife to go get and find more information, to find resources to support information to give women. (Midwife 1)

One midwife highlighted that continuing nutrition education should be a shared responsibility between midwives and their employers.

I think it's the responsibility of both the employer and the midwife herself to make sure that they're up to date with the knowledge that they need for their practice and…to keep abreast of…research that's coming out and changes in guidelines and those sorts of things. (Midwife 4)

When asked what guidelines or sources of information are available for midwives to provide nutrition advice, most midwives referred to written information they usually provided to pregnant women. Half the participants relied on guidelines and/or government resources (e.g., brochures, booklets, and websites) specific to the state or territory in which they worked. A few referred to national sources such as the National Health and Medical Research Council dietary guidelines and ACM or international guidelines or resources such as the National Institute for Health and Care Excellence guidelines.

Midwives lacked awareness of any guidelines or specific resources available especially for midwives to guide them in providing nutrition advice to pregnant women, with one midwife suggesting that academics should develop such guidelines or resources.

…as far as I know there's not actually any guidelines to guide midwives along nutrition….Certainly not in the midwifery guidelines. There's a vague mention of it in one of the elements in the midwifery practice guidelines but it's vague and not specific about how to teach or how to educate or how to get your information or anything else…. It's just saying that you should give good nutritional advice. (Midwife 12)

The reliability, validity, and layout of sources of information used seemed important to midwives, especially when using online and nongovernmental resources. Midwives commented that availability of the resources does not guarantee that midwives or women would access them.

Other sources of nutrition information were also mentioned by the participants, such as talking with dietitians, journal articles, resources provided by hospitals (e.g., handouts, and databases) or provided to hospitals in commercial packages (e.g., Bounty bag), and midwives' experiences.

Some midwives highlighted issues or problems with such guidelines and resources; for example, the written resources were often focused on food hazards more than health promotion and polices were slow to respond to change.

### 3.5. Barriers Midwives Encounter in the Provision of Nutrition Advice

The majority of the midwives, especially those involved in fragmented models of care, defined time constraints as one of the major barriers affecting their activities when it came to providing nutrition advice. Midwives in continuity of care models also commented on time constraints. From their perspective, midwives saw the public health system as providing limited time to engage with pregnant women in regard to nutrition, with some comparing their current privileged situation with previous experience in fragmented care.

Midwives commented that discussing nutrition took place usually at the booking visit, where the length of the visit was disproportional to the amount of work the midwife had to do and the amount of information the midwife had to convey to the women.

Well, I think time, if you work in a busy public hospital, time's the number one…they have to do so much. Like, for instance, in a booking interview…, at our hospital, when you see a woman for the first time it's an hour and a half interview and in that hour and a half you have to do so much, you've got to do their mental, physical, social history, psycho-social history, domestic violence screen, so many different things, and to throw nutrition into there, it's important but it's easily missed because of…all the paperwork, the documentation you have to do…. (Midwife 14)

One midwife highlighted that lack of time in public hospitals was not only an obstacle to provide the information to the women but also an obstacle for the midwife to update their knowledge, allocate resources, and access experts who can provide such resources. This in turn can lead to a passive approach when conveying nutrition information.

…in the public system everything is tightening up a lot. So time restricts you not only giving your patient care and having time for the education but it's also time for updating your practice, finding out new information, finding the brochures, accessing the dietitians and the people within the health system, the Allied Health people that could provide that information for you. So very much you tend to fob people off and sort of say, well you'll need to go and find that from there or you'll say well do an Internet search, you'll probably find that information there. (Midwife 12)

Midwives pointed out that lack of relevant and reliable nutrition education resources (such as handouts and web sites to refer women to or for the midwives to resource accurate information or continuing education from) may hinder their role in the provision of effective nutrition advice to pregnant women. This was particularly highlighted by midwives working with women from diverse linguistic and cultural backgrounds.

…it's not just because I work with Aboriginal women. I think it's working with women from culturally and linguistically diverse backgrounds, is that the actual resources that we have are very…targeted at middle class Caucasian women that have reasonable literacy skills…. And that's, having resources that aren't sort of targeted at a broader range group I think is part of the challenge…That would be from sort of a systems point of view. (Midwife 4)

Lack of nutrition knowledge was signalled by some midwives as a challenge to providing meaningful nutrition advice, especially lack of knowledge of other cultures' food choices.

…we get a lot of Asian women and African women in our community because…we have refugees that are relocated in our area. They actually struggle quite a lot. Well, we don't understand their culture anyway…. So their foods, because they are quite different to ours…I'm not sure how we would manage, cross that sort of cultural barrier. So certainly there's that barrier. (Midwife 12)

On the other hand, one midwife indicated that midwives are challenged by other sources of nutrition information, their reliability, and whether the mother trusts the midwives or those resources.

… As a midwife you're competing with other sources of information and you don't know whether they're valid or…you don't know how reliable they are and you also don't know where…the woman's choosing to put her trust… who she's going to…rely on. (Midwife 5)

Other constraints related to the health system identified by the midwives included the model of care (as previously described) and late encounter with pregnant women.

… I think the restrictions are the fact that we don't see them ‘til later in the pregnancy…Not ‘til about 13 weeks…well diet's important the whole way through but…that first trimester's already gone…. And you sort of wonder how they've been eating that whole time…by the end of the first trimester that baby's fully formed and just needs to grow so it's so important what they eat but we don't get hold of them until after that. (Midwife 3)

Many midwives commented that provision of the nutrition advice would be variable as it would be subject to midwives' attitudes, such as their interest in nutrition (or lack thereof), and their perception of its importance, and even their ability to be an appropriate role model.

The problem is…if people are going to be discussing nutrition with mums, there needs to be an intrinsic interest there…I'm really disappointed when I see midwives with higher BMIs [Body Mass Index] trying to give out dietary advice, so really that midwife can't take it herself so how can she be passing it on? (Midwife 11)

Approximately one in three of the midwives pointed out women's knowledge and attitudes as barriers to the provision of effective nutrition advice. “Lack of knowledge” referred to foundational knowledge provided by subjects taught in secondary education and lack of food-related skills (i.e., how to make healthy choices, shop, budget, and prepare a meal). Attitudes included lack of motivation to change dietary behaviour, lack of emphasis on diet compared to other issues (e.g., birth), body image (positive or negative), noncompliance (e.g., with gestational diabetes diet), guilty feelings, or fear of harming the baby.

Social determinants of health for the women, such as socioeconomic status, environment (i.e., access to healthy and affordable food, and access to allied health care services in remote areas), language and literacy, and culture were also barriers mentioned by the midwives as obstacles to the provision of effective nutrition advice to pregnant women.

The other thing is I think as midwives we are very conscious that sometimes the women that we're dealing with come from difficult circumstances so, you know, buying fresh fruit and vegetables might be difficult or cooking in your facility might be difficult, so you tend to opt for the easiest option and that often isn't the best in terms of nutrition…. That's where… the social determinants of health affect a midwife's ability to engage with a woman in regards to her nutrition…. (Midwife 1)

It was identified by a midwife that it is quite challenging to explain the concept of risk to women with lower education status as it may affect their reaction to the advice provided.

…we do talk about the concept of risk …. But many women have no antenatal care, do drink throughout their pregnancy, do smoke, baby's not breastfed, baby's put straight onto the bottle and that baby will still chart nicely along a growth chart, will still do OK at school, will still be born and look normal and be of a good weight and so that's a really difficult, I think that's a challenge…Because the concept of risk is so abstract…. I think the expectations we have of clients to really understand that is, at times might be a little bit unrealistic…. (Midwife 4)

Midwives also referred to women's family and work commitments or incorrect advice from relatives as hurdles affecting their provision of nutrition advice.

The other challenge we have is the grandmothers…And they're going… I ate whatever I felt like and you were fine so what's the problem?… (Midwife 2)

### 3.6. Facilitators That Would Help Midwives Provide Better Nutrition Advice

Midwives repeatedly commented that continuing education about nutrition issues or regular updates on the latest evidence-based nutrition information would be helpful for them to provide quality nutrition advice. However, they also stressed that continuing education or updates need to be provided by reliable sources such as the ACM and health services, as that would spare their time in navigating the abundant sources of information available. Online learning basic modules or courses and in-service education were frequently mentioned.

A common strategy suggested by the midwives was collaboration with allied health professionals, especially dietitians as they were perceived as the “experts” who had the training and the knowledge. Collaboration with the dietitians and other alternative or allied health professionals (e.g., naturopaths and physiotherapists) took many forms. For example, some midwives suggested that dietitians should be involved in developing useful resources such as handouts, DVDs, and websites that the midwives can then use to enhance their practice or reinforce the messages they provided to pregnant women.

Other participants proposed that dietitians educate the midwives and the midwives can then pass this education to pregnant women. It was suggested that dietitians need to be involved in maternity care by participating not only in antenatal classes (as it was indicated this might be late or may not capture all women) but also in antenatal appointments. One midwife even suggested the need for a permanent position for a dietitian in antenatal clinics, as regular hospital dietitians would not see pregnant women unless they had complex issues. This suggestion was based on her experience of having a temporary dietitian in her work place and how that was beneficial not only for pregnant women (who may need quick personal chats) but also for midwives who used talking with the dietitian as a source for up-to-date information. Having back-up dietitians in the community for pregnant women in the private sector was also recommended. However, it was realised that involving dietitians is a system issue that might involve time and cost.

…there should be the ability for midwives to be in collaboration with dietitians or nutritionists…I know that's time consuming and I know that it's often hard to negotiate that and… [it] costs money, however, I think that would be the best way to do it. (Midwife 12)

Another strategy to help in preparing midwifery students to provide better nutrition advice suggested by the participants was incorporating nutrition into midwifery education. Midwives also suggested specific attributes for this incorporation, including as an in-depth compulsory designated unit in all midwifery programmes; in a broad way involving varied health professionals such as dietitians and naturopaths; and in a practical manner that meets the needs of practice in the real world.

Midwives also recommended other strategies related to the provision of nutrition advice in the antenatal care context in general. This included better funding; standardisation of information; availability of resources that meet the needs of young women, such as special applications and web sites to enable early dissemination of nutrition information; and taking on a preventive approach rather than a management one. Other facilitating strategies included the availability of literacy appropriate resources; increasing the awareness of guidelines; and doing more research to improve nutrition education provided to pregnant women. A few midwives proposed that more time needed to be allocated to antenatal visits, so midwives could provide meaningful nutrition education.

Well, more time… at the end of the day if the midwives don't have time to talk to women about nutrition, I guess there's no point them resourcing the information…(Midwife 7)

## 4. Discussion

This qualitative study explored Australian midwives' perceptions of their role in providing nutrition advice, the effect of their model of care on this role, and what facilitated or hindered it. Midwives believed that they have a vital role in providing nutrition advice to pregnant women generally in the context of health promotion; however, this was not reflected in the advice many of them provided. The advice seemed in many accounts passive and more medically directed. Despite midwives' beliefs in their role in this area, the extent and efficacy of this role appeared to be challenged by many barriers. Midwives suggested some facilitators that may help overcome these challenges.

The perception of midwives in this study was that providing nutrition advice to pregnant women was a vital part of their role. This is consistent with previous international literature and the quantitative results of the cohort from which this sample was recruited [12, 16], especially in regard to providing holistic nutrition advice in the context of health promotion [10]. Our findings also support the argument of Cheyney and Moreno-Black [17] that midwives view food and nutrition as an integral part of the holistic care that underpins their midwifery model of care and as a means to maintain health and wellbeing. Similarly midwives in this study referred to viewing and using food as the “first medicine” and as part of the holistic care they provided to pregnant women.

Despite midwives' belief in their role in providing general nutrition advice in the context of health promotion, this was not reflected in the generalised or passive approach many midwives in this study utilised in the way they conveyed nutrition information to pregnant women. They expressed a tendency to rely on written information as a means for providing advice, particularly for women perceived and assumed to be healthy. These findings reflect the finding of Bondarianzadeh et al. [18] who found that Australian midwives have an overreliance on written information and adopt mostly a passive approach when advising pregnant women about listeria.

Midwives' advice in this study was on many accounts medically directed or provided when suspecting a deficiency or managing an issue. These results further confirm the quantitative findings of this cohort [12] and concur with international research that has reported pregnant women received nutrition advice from midwives only when there were health issues or concerns [19]. Some midwives in this study refrained from or adopted a passive approach in discussing certain topics with pregnant women when they perceived them to be motivated and educated. While such an approach may be considered to have merit, it is not based on evidence. Bookari et al. [20] found that a sample of mostly highly educated and motivated Australian pregnant women had poor nutrition knowledge and dietary behaviour and suggested health care providers should not base their provision of nutrition advice to those women on such assumptions.

The midwives' accounts of the barriers and the challenges that restrict the midwife's role in providing effective nutrition advice could be considered to be related to the health system or otherwise out of their control, citing issues such as lack of time, lack of resources, limited basic and continuing nutrition education, and the effect of their model of care. These results are in line with earlier literature where insufficient time, resources, and education were considered the main obstacles that impeded health care providers from educating pregnant women about nutrition [8].

The midwifery philosophy is centred on building a relationship with the women and supporting and empowering them to make informed decisions that will improve their health and ultimately the health of the society [21]. This relationship forms the basis for establishing rapport, trust, respect, and confidence with the women which were the pillars of effective nutrition communication according to most midwives in this study. This was especially highlighted by the remarks of most midwives providing continuity of care. Lack of time to establish a relationship was frequently referred to as a barrier by the midwives (mostly in fragmented care) to their engaging with pregnant women in discussing nutrition issues, especially sensitive topics such as weight gain and obesity, which is consistent with previous studies [22]. Motivational interviewing has been suggested as an effective strategy that can be used by midwives when approaching women with regard to sensitive issues such as obesity or to overcome barriers related to the women, such as their attitudes, for example [23]. Several participants also suggested nutrition or health promotion approaches may be effective but there was little indication that they were informed about what such approaches may involve or whether they had the necessary skill set for such an approach.

Midwives suggested a number of facilitators to overcome the barriers that challenged their role in providing nutrition advice. Midwives requested continuing education in general nutrition. This may reflect their perceptions of the advice they needed to provide as “general.” They requested more education from reliable sources to feel supported and to reduce the overreliance on SDL that was perceived to be dependent on time availability and midwives' interest. They also requested collaboration with health professionals they considered the “experts.” While such continuing education may be helpful in relation to the nutrition content, midwives did not identify the need for in-service education in relation to the skills necessary to promote nutrition to pregnant women or support their attempts to change their diets.

Midwives in this study commented that they mainly relied on state-specific guidelines to guide their practice in providing nutrition advice but highlighted an absence of midwifery guidelines. National clinical guidelines have been developed in Australia since this study was undertaken [24, 25]. These guidelines specify that antenatal health care providers, including midwives, need to discuss nutrition at every antenatal opportunity by highlighting its importance, assessing maternal diets, and providing advice in a holistic approach considering social determinants of health and referral to dietitians where appropriate. However, a recent Australian study examining women's perceptions of antenatal care provided to them against those guidelines found weight gain and diet to be the areas raised the least [26]. Midwives would need support on many levels if they are to discuss nutrition in an individualised and holistic approach, as specified by the current clinical guidelines. Intervention studies are needed to ascertain the best strategies to help midwives and other health care providers incorporate nutrition advice effectively into their practice and overcome common barriers.

### 4.1. Limitations and Strengths

The nature of qualitative research limits its generalisability; however, this qualitative study enabled more understanding of midwives' perceptions of their role in advising women about nutrition and the challenges that restricted this role. Even though only 16 midwives participated, they were from both public and private sectors and were engaged in various models of care. Saturation of data was reached at 16 interviews as no new ideas emerged. The majority of the participants were of mature age with less representation from younger midwives, reflecting the age profile of the midwifery workforce in 2012 [27].

### 4.2. Future Research

Future research needs to involve interviewing younger or newly graduated midwives to explore their perceptions of their role in providing nutrition advice during pregnancy and their views of their educational preparations to provide such advice to cite any differences in this regard from the views of mature midwives interviewed in this study.

## 5. Conclusion

Although midwives in this study perceived providing nutrition advice to pregnant women as an integral part of their practice as midwives, this role was felt to be constrained by many challenges and factors mostly out of the midwives' control. Midwives need structured assistance, support, and guidance to provide holistic nutrition advice that assists women to achieve healthy pregnancies. Changes in the policy of health care services were suggested such as allowing more time for antenatal visits; encouraging continuity of care for all women; creating permanent positions for dietitians in antenatal clinics; and developing free online nutrition models and training packages for practising midwives by the professional organisations. A collaborative approach between midwifery bodies, nutrition, and education experts and maternity care services should be considered to implement such changes. While this study was confined to Australia, the findings and recommendations have relevance to other countries that support midwifery services.

## Figures and Tables

**Table 1:** Midwives' specified models of care.

Midwife number	Models of care as specified by the midwives
Midwife 1	The team leader, midwifery-led model of care/continuity of care
Midwife 2	Lactation consultant/also has a private practice/does antenatal care but works mostly in postnatal care
Midwife 3	Caseload/continuity of care
Midwife 4	Community-based model/continuity of care
Midwife 5	Hospital/rotate through different areas and does antenatal clinic/fragmented care/had been doing antenatal care for a few months
Midwife 6	Private hospital/see women at booking only
Midwife 7	Midwifery-led care/continuity of care
Midwife 8	Hospital/midwifery educator/does antenatal clinic/fragmented care
Midwife 9	Private practice for an obstetrician/fragmented care
Midwife 10	Hospital/rotate through different areas/fragmented care/also a clinical midwife for midwifery model of care for Aboriginal and Torres Strait Islander women
Midwife 11	Public hospital/fragmented care/mainly antenatal care in an Aboriginal health clinic and a midwives' clinic in the country/also has private practice
Midwife 12	Community-based/continuity of care
Midwife 13	Team midwifery/birth centre/continuity of care
Midwife 14	Hospital/educator/does antenatal classes/used to work in clinical area
Midwife 15	Multidisciplinary team of midwives with residents and consultants/rotate through the clinic/fragmented care
Midwife 16	Midwifery Group Practice (Caseload)/continuity of care
